# Healthy Growth in Children with Down Syndrome

**DOI:** 10.1371/journal.pone.0031079

**Published:** 2012-02-17

**Authors:** Helma B. M. Van Gameren-Oosterom, Paula Van Dommelen, Anne Marie Oudesluys-Murphy, Simone E. Buitendijk, Stef Van Buuren, Jacobus P. Van Wouwe

**Affiliations:** 1 Department of Child Health, Netherlands Organization for Applied Scientific Research (TNO), Leiden, The Netherlands; 2 Department of Life Style, Netherlands Organization for Applied Scientific Research (TNO), Leiden, The Netherlands; 3 Department of Pediatrics, Leiden University Medical Centre, Leiden, The Netherlands; 4 Leiden University Medical Centre, Leiden, The Netherlands; 5 Department of Methodology and Statistics, FSS, University of Utrecht, Utrecht, The Netherlands; Nathan Kline Institute and New York University School of Medicine, United States of America

## Abstract

**Objective:**

To provide cross-sectional height and head circumference (HC) references for healthy Dutch children with Down syndrome (DS), while considering the influence of concomitant disorders on their growth, and to compare growth between children with DS and children from the general population.

**Study design:**

Longitudinal growth and medical data were retrospectively collected from medical records in 25 of the 30 regional hospital-based outpatient clinics for children with DS in the Netherlands. Children with Trisomy 21 karyotype of Dutch descent born after 1982 were included. The LMS method was applied to fit growth references.

**Results:**

We enrolled 1,596 children, and collected 10,558 measurements for height and 1,778 for HC. Children with DS without concomitant disorders (otherwise healthy children) and those suffering only from mild congenital heart defects showed similar growth patterns. The established growth charts, based on all measurements of these two groups, demonstrate the three age periods when height differences between children with and without DS increase: during pregnancy, during the first three years of life, and during puberty. This growth pattern results in a mean final height of 163.4 cm in boys and 151.8 cm in girls (−2.9 standard deviation (SD) and −3.0 SD on general Dutch charts, respectively). Mean HC (0 to 15 months) was 2 SD less than in the general Dutch population. The charts are available at www.tno.nl/growth.

**Conclusions:**

Height and HC references showed that growth retardation in otherwise healthy children with DS meanly occurs in three critical periods of growth, resulting in shorter final stature and smaller HC than the general Dutch population shows. With these references, health care professionals can optimize their preventive care: monitoring growth of individual children with DS optimal, so that growth retarding comorbidities can be identified early, and focusing on the critical age periods to establish ways to optimize growth.

## Introduction

Appropriate, up-to-date growth charts are necessary for evaluation of physical growth and provision of optimal health care. The World Health Organization has produced a global standard chart describing how children, under optimal conditions, grow worldwide. [1] This is based on the idea that all humans are more or less equal. Health care workers, on the other hand, often wish to use growth charts of a well defined reference group closely related to the subpopulation they serve, since these charts provide a more accurate evaluation for an individual child. [2] Growth charts are available for various ethnic groups at specific moments in time. [3], [4] Specific growth references have also been developed for children with various disorders known to interfere with growth, such as Turner and Down syndrome (DS). [5]–[9] Since growth assessment depends on the growth pattern characteristic for these conditions, disorder specific charts are desirable. Growth references for American children with DS have been constructed making it possible to accurately identify concomitant disorders known to influence growth. [6] Growth charts for Dutch children with DS were first published in 1996: they are shorter than children in the general Dutch population, but taller than their US peers with DS. [8]

In order to take the secular trend into account, growth references for height and head circumference (HC) need to be updated regularly. [3] Children with DS are at high risk of many disorders known to influence growth. Such disorders are generally regarded as exclusion criteria in growth studies: all children diagnosed with growth disorders or on medication known to interfere with growth are usually excluded. [3] However, in studies on growth in children with DS such exclusion criteria are usually not applied. [5]–[8], [10] Only two recent growth studies in children with DS (in Japan and in the UK and Ireland) excluded children with various diagnoses known to affect growth. [11], [12] In addition, no previous studies have investigated in which particular age periods height growth in otherwise healthy children with DS is relatively most delayed, by comparing their growth with that of healthy controls from the general population.

Therefore, the aim of the present study is to provide updated height and new HC growth references by a large nationwide sample, reflecting healthy growth in Dutch children with DS, and to compare their growth pattern with data from a recent nationwide study among children from the general Dutch population with focus on periods during which relative height differences increases. We think it is essential to establish new growth references for children with DS in the Netherlands, whereby a strict selection on their health status will be applied. Only with such references health care professionals can monitor growth of individual children with DS optimally, and can identify growth retarding comorbidities at an early stage.

## Methods

### Data source

To collect representative nationwide data, all specialized regional pediatric outpatient clinics for children with DS in the Netherlands were approached (n = 30). These hospital-based clinics provide standard medical care for children with DS, according to the guideline of the Pediatric Association of the Netherlands. [13] All children with DS, who live in the service area of these clinics, are eligible to participate in this standard care by specialized pediatricians that includes screening for congenital cardiac defects, thyroid dysfunction, celiac disease, hearing and visual disorders as well as motor development and growth monitoring. Youth health care physicians working in special education and looking after older children with DS were also approached. Names of all participating clinics, pediatricians and youth health care physicians are mentioned in the acknowledgement.

At the clinics all data were retrospectively collected from medical records, between July 2009 and February 2010, by the first author, who is a trained physician. The de-identification was also completed by the first author, using study numbers. Additional data from the youth health care physicians caring for older children with DS in special education were collected by completing standard forms. All measurements were carried out according to protocol. [14] Height was measured using a recumbent length device or a stadiometer and HC by using a measuring tape (fiberglass or other non-expanding material). Data concerning medical conditions and treatment of each subject, and specifically those conditions known to interfere with growth, were gathered from medical records, as well as specific background information. Children were considered to be of Dutch descent if both parents were born in the Netherlands, as reported in the medical records. If medical or background information was not available, subjects were excluded.

### Inclusion/exclusion

Children with Trisomy 21 karyotype were selected, and those with DS caused by mozaïcisme or translocation were excluded (verified by karyotype). All children born after 1982 were included (aged up to 26 years). We collected semi-longitudinal data on growth over the previous 10 years, recording data from 2000 onwards. We selected only the first recorded observation per interval: from an infant (age of 0 to 1 year old) one observation in each month, from a toddler (age 1 to 3 years old) one observation in each three months period, and one observation in each six months in childhood and adolescence (age up to 26 years old). Measurements of HC were selected only from full-term children (born ≥37 weeks gestation) up to the age of 5 year. If gestational age was not specifically mentioned, children were considered to be born at term.

### Health status

The children were categorized according to their health status in 4 groups: healthy, with only mild CHD, with only severe CHD or with other (multiple) concomitant disorders. Table 1 describes the characteristics of the various health categories and details of the criteria used for these categories.

**Table 1:** Characteristics of the various health categories in the study population of children with Down syndrome.

**Healthy**
Children without concomitant disorders that could possibly interfere with growth
Children were negatively screened for CHD, celiac disease and hypothyroidism
For example: children with cataract were included and children with musculoskeletal disorders were excluded
**Mild CHD**
Children with CHD not needing surgical intervention or medication and without pulmonary vascular disease
For example: children with an atrial septal defect or patent foramen ovale without complaints
**Severe CHD**
Children with CHD needing surgical intervention, medication, or with pulmonary vascular disease
For example: children with an atrioventricular septal defect or Tetralogy of Fallot
**Other disorders**
Children with other disorders and treatments known to interfere with growth, and children with multiple concomitant disorders
For example: children positively screened for hypothyroidism or celiac disease, children with congenital gastrointestinal disorders, children on anti-epileptic medication or corticosteroids (including inhalation medication)

Abbreviation: CHD – congenital heart defect.

Permission of an Ethical Committee or informed consent is not obtained, as not required in the Netherlands for this type of study or data collection, since the data were analyzed anonymously.

### Statistical Analysis

Data cleansing was performed by excluding duplicate cases and outliers. Duplicate cases have arisen when children were seen at multiple centers and were identified by comparing sex, date of birth and background information such as nationality, and medical condition. Outliers were defined using height and HC standardized by age and sex according to the reference charts of the general Dutch population, calculated in standard deviation scores (SDS). [3] Cutoff values >2 or <−6 for height SDS and HC SDS were used. If a child had one measurement outside the cutoff values, all measurements of this child were excluded. Moreover, the longitudinal growth pattern of each subject was checked by plotting them and excluding values outlying the plot. Data were analyzed separately for boys and girls.

The difference in mean height SDS and HC SDS between healthy children with DS and children with DS and a mild or severe CHD were tested by linear mixed-effects models. To correct for possible differences in the prevalence rates of sex and mean age, we adjusted these analyses by age and sex. Furthermore, growth references for boys and girls with DS were fitted for body height by age (range 0 to 21 years) and HC by age (range 0 to 15 months) using the LMS method. Growth references were fitted in R Version 2.9.0 using Generalized Additive Models for Location Scale and Shape (GAMLSS). [15] The LMS method summarizes the distribution by three age-dependent smooth curves representing skewness (L curve), median (M curve) and coefficient of variation (S curve). [16] The LMS method is based on the principle that after a transformation the data have to follow a standard normal distribution. The following transformations of age were tested to expand the ages where growth velocity is high and compress age where growth velocity is low: the age transformation proposed by Cole, a square root transformation, a log transformation and a cube root transformation. [17] Worm plots were used as a diagnostic tool to visualize the fit to the data. [18] These plots check the residuals for different age levels and indentify locations at which the fit can be improved. We fitted charts with lines of the −2.5, −2.0,−1.0, 0, 1.0, 2.0 and 2.5 SD (corresponding with 0.6, 2.3, 15.9, 50, 84.1, 97.7 and 99.4 percentile). The choice of these SD-lines is in agreement with the latest reference charts for the general Dutch population. We did not fit growth charts for the health category ‘other disorders’, because of the wide variety of data and medical conditions of the children in this category. Also, growth charts for subgroups in this category – like children with hypothyroidism or celiac disease – were not fitted, because of the small number of children included in our study.

In addition to the reference charts for height, formulas for Target Height (TH) for children with DS were calculated. [19] The TH was calculated by the method of Hermanussen and Cole, which takes into account two correlations (assortative mating r(P,P) and the parent-offspring correlation r(P,O)). [20] For this calculation, the data from three nationwide sources have been applied. The correlations were obtained from the latest growth study in the general Dutch population (2009) and were r(P,P) = 0.19 and r(P,O) = 0.58. [21] The calculation for the paternal and maternal height SD was based on the results from the previous national growth study (1997); final height of children measured in this study corresponds with final height of this generation of parents. [3] For the calculation of the TH SDS the mean and SD of final height of the children with DS observed in this study were used.

The established growth references for height and HC were compared with the most recent reference charts for the general Dutch population, by calculating SDS. [21]

## Results

Pediatricians working in 25 hospital-based clinics (83% of all Dutch DS clinics) and 14 youth health care physicians caring for older children with DS in special education agreed to participate in this study. After exclusion of 7 children with a growth pattern outside the cut-off values, a total of 1,843 children with Trisomy 21 were indentified. Of these, 1,596 are of Dutch origin: 891 boys (55.8%) and 705 girls (44.2%). The sample provided 10,558 measurements for height and 1,778 for HC in the age range 0 to 5 years and born at term (418 HC measurements from children born pre-term in this age range were excluded). Of all height measurements, 98% were derived from the DS clinics and 2% from the youth health care physicians in special education. The HC measurements were only derived from the DS clinics. Table 2 provides total number of subjects and measurements of height and HC, split according to sex and to various health categories. In our sample, 26.6% was categorized as healthy, 15.0% had only a mild CHD and 16.9% only a severe CHD. The remaining group, 41.5%, was categorized as having various other disorders; most of them (over 60%) had multiple concomitant disorders.

**Table 2:** Number of subjects and measurements for height and head circumference of 1,596 Dutch subjects with Trisomy 21, specified by the health categories.

	Subjects				Measurements			
	Height		HC^1^		Height		HC	
Health category	Boys	Girls	Boys	Girls	Boys	Girls	Boys	Girls
Healthy	257	167	71	57	1,378	938	202	139
Mild CHD	130	110	49	46	751	661	169	147
Severe CHD	114	155	38	50	738	997	134	187
Other disorders	390	273	140	88	2,970	2,125	443	357
Total	891	705	298	241	5,837	4,721	948	830

Abbreviations: HC – head circumference, CHD – congenital heart defect.

1 Only children born full-term up to the age of 5 years were included.

### Growth of children with DS within various health categories

Growth of healthy children with DS and those who suffer from mild CHD showed no difference in mean height SDS (p = 0.832) and HC SDS (p = 0.790). Both girls and boys with DS and severe CHD had significant lower mean height SDS and HC SDS compared to healthy children with DS or children with DS with mild CHD (both p-values<0.001). Mean height is observed to be 0.4 SD lower. This growth retardation arises in the first year of life; during childhood no further deflection neither catch-up growth was observed.

### Growth references

Data for growth references of height and HC were derived by combining the groups of healthy children with DS and children with DS and mild CHD (664 children for height, and 223 for HC (see Table 2). The number of measurements of these selected groups, arranged by age en sex, are shown in Table 3. By fitting the growth references for height, the cube root transformation of age showed the best fit and was selected. Table 4 summarizes mean height and SD, arranged by age and sex. Mean birth length was 48.9 cm in boys and 48.4 cm in girls with DS. Mean final height was 163.4 cm in boys and 151.8 cm in girls. These new reference charts are available at www.tno.nl/growthhttp://www.tno.nl/growth.

**Table 3:** Frequencies of measurements for height used for plotting the reference curves for Dutch children with Down syndrome, arranged by age and sex.

	Height^1^		
Age (years)	Male	Female	Total
0	474	392	866
1	241	201	442
2	201	147	348
3	178	129	307
4	151	101	252
5	132	96	228
6	117	86	203
7	83	89	172
8	105	66	171
9	86	49	135
10	72	52	124
11	62	46	108
12	68	37	105
13	52	29	81
14	36	28	64
15	31	12	43
16	20	14	34
17	8	6	14
≥18	12	19	31
Total	2129	1599	3728

	Head circumference^2^		
Age (years)	Male	Female	Total
0	244	199	443
1	69	50	119
2	22	18	40
3	19	10	29
4	17	9	26
Total	371	286	657

1 Height measurements of the children categorized as healthy or with only mild CHD (n = 664).

2 Head circumference of the children categorized as healthy or with only mild CHD, born at term (n = 223).

**Table 4:** Mean height (cm) and standard deviation (SD) of the new references for length/height of Dutch children with Down syndrome, arranged by age and sex.

	Boys		Girls	
Age (weeks)	Mean	SD*	Mean	SD*
0	48.9	2.5	48.4	2.1
2	50.4	2.6	49.8	2.2
4	51.9	2.6	51.3	2.2
6	53.4	2.6	52.6	2.2
8	54.8	2.6	54.0	2.2
10	56.2	2.6	55.3	2.2
12	57.5	2.6	56.5	2.3
16	60.0	2.6	58.7	2.3
20	62.2	2.6	60.7	2.3
24	64.1	2.7	62.6	2.4
28	65.8	2.7	64.2	2.4
32	67.3	2.7	65.7	2.4
36	68.6	2.7	67.0	2.5
40	69.8	2.7	68.2	2.5
44	70.9	2.8	69.3	2.5
48	71.9	2.8	70.3	2.6
52	73.0	2.8	71.3	2.6
Age (years)				
1.5	78.3	3.0	76.4	2.8
2.0	82.6	3.1	80.8	3.0
2.5	86.4	3.3	84.6	3.2
3.0	89.8	3.5	88.1	3.5
3.5	93.1	3.7	91.6	3.7
4.0	96.4	3.9	95.2	3.9
4.5	99.7	4.0	98.6	4.1
5.0	102.9	4.2	101.7	4.3
6.0	109.0	4.5	107.2	4.6
7.0	114.7	4.8	113.0	4.9
8.0	119.9	5.0	118.7	5.2
9.0	125.2	5.3	124.0	5.5
10.0	130.8	5.5	129.3	5.8
11.0	136.8	5.7	134.4	6.1
12.0	142.9	5.8	139.1	6.3
13.0	148.7	5.9	142.9	6.5
14.0	153.5	6.0	145.9	6.6
15.0	157.2	6.1	147.9	6.8
16.0	159.8	6.2	149.4	6.9
17.0	161.8	6.2	150.5	7.0
18.0	163.0	6.2	151.2	7.1
19.0	163.4	6.2	151.6	7.2
20.0	163.4	6.2	151.8	7.3
21.0	163.4	6.2	151.8	7.3

*Individual height SDS can be calculated by: SDS = (height (cm)−mean height (cm))/SD (cm).

Abbreviations: SD – standard deviation, SDS – standard deviation score.

Growth references for HC were established for up to the age of 15 months. We did not have enough measurements to construct growth references after the age of 15 months. By fitting HC references for boys no transformation of age and for girls the age transformation proposed by Cole was applied. [17] Table 5 summarizes the LMS-values, arranged by age and sex. At birth mean HC was 33.8 cm in boys and 32.9 cm in girls with DS; at the age of 15 months this was 45.0 cm and 43.7 cm respectively.

**Table 5:** New head circumference (cm) references for 0–15 months in Dutch children with Down syndrome: values of L (skewness), M (median), and S (coefficient of variation)*, categorized by age and sex.

	Boys			Girls		
Age (weeks)	L	M	S*	L	M	S*
0	2.96	33.8	.0349	1	32.9	.0267
2	2.65	34.6	.0343	1	33.8	.0272
4	2.34	35.3	.0336	1	34.7	.0277
6	2.03	36.1	.0330	1	35.5	.0280
8	1.72	36.8	.0324	1	36.4	.0281
10	1.42	37.5	.0319	1	37.1	.0279
12	1.12	38.2	.0313	1	37.8	.0276
16	0.54	39.4	.0302	1	38.8	.0270
20	−0.01	40.4	.0292	1	39.7	.0265
24	−0.52	41.2	.0283	1	40.4	.0261
28	−0.99	42.0	.0274	1	41.0	.0259
32	−1.41	42.6	.0266	1	41.5	.0257
36	−1.80	43.1	.0258	1	42.0	.0255
40	−2.14	43.5	.0250	1	42.3	.0254
44	−2.45	43.9	.0243	1	42.7	.0252
48	−2.72	44.2	.0237	1	42.9	.0251
52	−2.97	44.4	.0231	1	43.2	.0249
56	−3.19	44.6	.0225	1	43.3	.0248
60	−3.40	44.8	.0221	1	43.5	.0247
65^a^	−3.64	45.0	.0216	1	43.7	.0245

a Corresponding with 15 months.

*Individual head circumference SDS can be calculated by: SDS = {(height (cm)/M)^L^−1}/L*S, L≠0 (if L = 0: SDS = ln(height (cm)/M)/S).

### Target Height

The calculated formulas for TH of children with DS are:
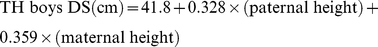


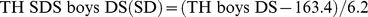


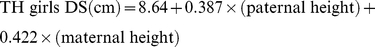


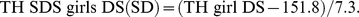



### Comparison to the general Dutch population

**Figure 1:**
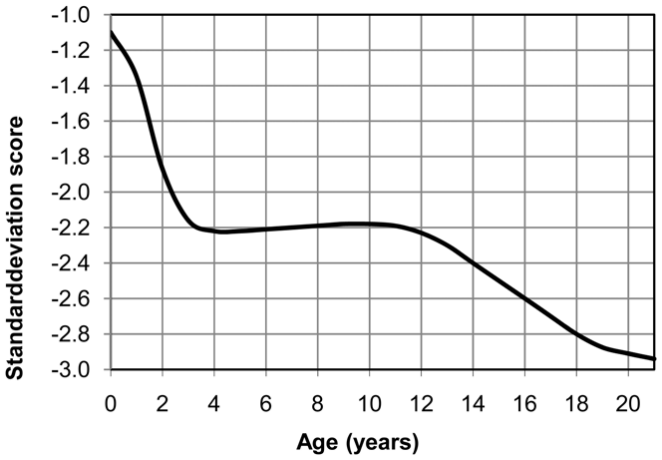
Mean height (SDS) of the Dutch children with Down syndrome compared to the general population. Comparison is made to the mean height of the national Dutch growth study (2009), calculated in standard deviation score (SDS).

In comparison with the currently used reference charts for the general Dutch population, a markedly shorter stature was found in children with DS. [21] Mean height revealed a deflection in the first 3 years of life from −1.1 SD at birth to −2.2 SD at 3 years, for boys as well as girls. During the age interval of 3 to 12 years average growth remained stable at −2.2 SD, and deflected again during puberty to a final height at −2.9 SD (20.4 cm shorter) for boys and −3.0 SD (18.9 cm shorter) for girls (Figure 1). [21] For HC, between birth and 15 months, mean values for boys and girls with DS are on average 1.8 SD lower (range −1.3 to −2.0) compared to the general Dutch population. [3]

## Discussion

This study yields new charts of healthy growth of children with DS. Growth patterns were analyzed in 1,596 Dutch children with DS, with 10,558 height measurements and 1,778 HC measurements. All children were selected nationwide from pediatric hospital-based outpatient clinics specialized in the standard care of children with DS. Selection based on health complaints or influenced by financial restrictions was avoided, since all children with DS in the Netherlands were invited to avail of this care without financial barriers, as encouraged by the Dutch Down Syndrome Foundation (largest Dutch parent supporting organization). The result is a nationwide representative sample.

Our new reference charts for Dutch children with DS are unique. No previous studies on growth in DS made such a stringent selection of subjects with respect to their health. All children have received high quality medical care according to the guideline of the Pediatric Association of the Netherlands and underwent standard screening at regular intervals, provided by the regional hospital-based outpatient clinics for children with DS. [13] This screening programme provided confidence that children categorized as healthy in fact have no undiagnosed concomitant disorders, such as hypothyroidism, mild CHD and celiac disease. To illustrate the stringent selection: children with positive screening results for hypothyroidism and celiac disease were excluded (even though appropriate treatment was started early), because there is no evidence that growth could not be affected already.

The children with severe CHD showed growth retardation during the first year of life. After this period they had growth velocities similar to the healthy children with DS. In the Netherlands the surgical correction of significant CHD takes place preferably at the age of 2–4 months. [22] This early treatment may result in normal growth after the age of 1 year without further deflection. Cronk et al.also described growth retardation caused by CHD in children with DS. [6] However, they were unable to evaluate the effects of different types of cardiac lesions (those which healed spontaneously or those treated by corrective surgery) on growth. The present study provided appropriate data to make this distinction.

When comparing mean final heights of children with DS with other countries, we should take into account the different inclusion and exclusion criteria (Table 6). However, it is probably reasonable to conclude that Dutch children with DS are relatively tall among children with DS. Growth charts are available for West-European children with DS in Sweden, the UK and Ireland, and Northeastern France. [5], [12], [23]

**Table 6:** Growth studies presenting mean final height (cm) of children with Down syndrome, with the applied inclusion and exclusion criteria, by country of origin.

		Inclusion	Exclusion criteria	Boys	Girls
The Netherlands	This study	T21	All concomitant disorders known to interfere with growth; separate analyses of mild and severe CHD	163.4	151.8
Sweden [5]	2002	DS	Treatment with growth hormone	161.5	147.5
UK, Ireland [12]	2002	DS	Coexistent major pathology such as severe CHD or preterm birth	157	146
Japan [11]	2003	T21	Complications that might affect natural growth	153.2	141.9
France [23]	1999	T21	Severe CHD	154	140
USA [6]	1988	T21	Separate analyses of moderate and severe CHD (if information is available)	153	146

Abbreviations: T21 – Trisomy 21, DS – Down syndrome (including DS caused by Trisomy 21, mozaïcisme or translocation), CHD – congenital heart defect.

Our study is the first to propose formulas to estimate the expected final height in children with DS. The TH formulas were derived under the assumption that the correlation between mid parental height SD and child height SD in Dutch children with DS is identical to the general Dutch population. Further research is needed to investigate whether this assumption is justified.

We compare HC in our population with values published by others. Reference charts for HC are also available for children with DS from Sweden, Northeastern France, Sicily, UK and Ireland, the USA, Egypt and Saudi Arabia, suitable for use in these countries. [5], [10], [12], [23]–[26] Benefits of our study are the LMS-values presented, whereby for each individual the deviation exactly can be calculated. Comparing HC showed the Swedish children with DS have an average HC of 33 cm at birth, similar to our children with DS. [5] This mean values at birth corresponds with −0.5 SD on the reference charts for the general Swedish population, however with −1.5 SD for the general Dutch population. At 15 months mean HC is also identical in Swedish and Dutch children with DS. Since the other studies do not present enough details of their observations, we were unable to make further comparisons.

What are the main differences in growth pattern between DS and the general population? Mean birth length in DS is 1 SD lower than in the general population, indicating that children with DS already show retarded growth during pregnancy. Also, during the first three years of life they grow slower compared to the general population; the gap stays relatively constant during the age interval 3–12 years. After the age of 12 year a further deflection in growth is observed. This pattern is observed to be the same in boys and girls with DS. The mentioned three periods are already described by Karlberg et al. in the infant-childhood-puberty (ICP) model, which described during infancy (from birth up to about 3 years of age) and during puberty higher growth velocity than during childhood. [27] So, the children with DS show growth retardation just during the critical periods of growth when the highest growth velocity occurs. This finding indicate the age periods in which further health benefit may be obtained. Health care professionals should focus on these critical periods when providing preventive care to children with DS with the aim to establish ways to optimize growth during these specific periods. Either the observed growth retardation may be due to their genetic makeup or it may be caused by physical problems they encounter, such as feeding problems. In puberty an early or short growth spurt also limits final growth. Further research is needed to explain our observations and to provide physiological clarification on the nature of this growth retardation. All in all, these effects result in a substantial difference in final height (boys: 20.4 cm; girls: 18.9 cm).

Body weight will be reported separately. Because of the current increase in the proportion of children with obesity, it is not desirable to reflect the present distribution of weight in this population. Therefore, the increase in the proportion of children with obesity and the need for normative charts for weight deserve special attention. [21], [28]

A limitation of our study is that the measurements of height and HC were retrospectively collected. This methodology may lead to more variation in the measurements compared to studies that used prospectively collected data, as all general Dutch growth studies did. We were however unable to detect such increased measurement variability in the data. The S-curves (which model the coefficient of variation) were quite similar in our growth charts and the charts of the general Dutch population (established in 2009). So, we do not expect that the used methodology had an impact on the variability in the growth charts. A benefit of the applied methodology is the larger and timelier data set. Similar methods were used in other growth studies in children with DS. [5], [12], [23]

The longitudinal data resulted in a varied number of measurements per child. To determine the possible influence of this variation, the statistical analyses were repeated where data points were weighted – whereby a weighting factor was calculated as the inverse of the number of measurements per child – in order to prevent over-representing children with a large number of measurements. This solution was almost the same as the unweighted analysis; differences in mean height were smaller than 0.1 SD.

For the interpretation of individual growth curves plotted on a reference chart, criteria are needed to define abnormal growth. No specific criteria for the charts for children with DS have been proposed. The utility of the referral criteria for the general Dutch population as presented in the guideline ‘Detection and referral criteria in short stature’ has not been tested for growth in children with DS. Further research is necessary to see whether such referral criteria are equally suitable for children with DS. [29], [30] For the moment, we tentatively suggest to use the criteria for the general population (which are all framed in SDS) for children with DS.

### Implications

The availability of appropriate up-to-date growth charts, that reflect healthy growth of height and HC in Dutch children with DS, will potentially improve the medical care they receive. Using these charts secondary growth abnormalities may be detected more accurately. For example, limited growth of height may be a symptom of hypothyroidism and a relative large HC may be caused by hydrocephalus. The charts were based on a large sample that is stringently selected on the basis of their health status, and therefore we will encourage research to investigate the suitability of these charts for international application.

In children with DS with severe CHD growth is decelerated during the first year of life, in comparison to reference growth in the healthy infants with DS. During childhood no catch-up growth is noticed in these children with DS and severe CHD, neither does their growth decline further. Future research should focus on the exact qualities of the observed deflections in growth of otherwise healthy children with DS: is their growth spurt restricted with a lower velocity or do other phenomena play a role. Lack of significant catch up growth in children with DS and severe CHD could be the result of similar failure to spurt. We can only hypothesis on the full nature of growth retardation in otherwise healthy children with DS: as their growth is primarily restricted by Trisomy 21 the mechanism is unknown. Is it primarily metabolic, hormonal or chondrocyte dysfunction? Or could it be explained by further advanced genome-wide analysis?

### Conclusions

Growth patterns in otherwise healthy Dutch children with DS were established based on data from a large nationwide population. Growth in healthy children with DS differ from children with DS and severe CHD (0.4 SD). The established growth charts demonstrate the three age periods when height differences between children with and without DS increase: during pregnancy, during the first three years of life, and during puberty. This growth pattern results in a mean final height of 163.4 cm in healthy boys with DS and 151.8 in girls (a difference of 3 SD in comparison to the general population), and mean HC at birth of 33 cm and 44 cm at the age of 15 months (almost 2 SD less than in the general Dutch population). All in all, with these new growth charts, that reflect healthy growth in children with DS, health care professionals can monitor growth of individual children with DS optimal. In this way, early identification of growth retarding comorbidities will be enabled and ultimately the health of children with DS will be improved.
